# Observations on the Influence of Religion upon the Health and Physical Welfare of Mankind

**Published:** 1837-07

**Authors:** 


### ART. III.

*Observations on the Influence of Religion upon the Health and Physical Welfare of Mankind.* By AMARIAH BRIGHAM, M.D.—*Boston,* 1835. 8vo. pp. 331.

*Remarks on the Influence of Mental Cultivation and Mental Excitement upon Health.* By AMARIAH BRIGHAM, M.D.—*Boston,* 1833. 8vo. pp. 130.

We look upon the object of both these publications, each of which we have read with much gratification, to be essentially the same; and it is an object of much importance. From various causes, which we must leave to the moral philosopher to develop, there appears to be a tendency, in various states and degrees of civilization, to over-exert the brain and nervous system, either in acts of intellect or emotion; and the condition of the most civilized portions of America and that of the most refined countries of Europe, diverse as they are, exhibit these over-exertions, each according to the actual position and circumstances of the community. In Europe, it is the intellect which is chiefly over-tasked, and in America the same fault exists in a still greater degree; whilst the emotions, in one direction at least, are also generally and excessively over-wrought. The struggles for maintaining existence, and the graspings of ambition call, in Europe, for exertions under which the energies of the brain give way. The peculiar circumstances of a new country, not yet abounding in social and refined enjoyments, a country vast in extent, and grand in its features, and a new community, where every thing is to be obtained by talent and industry, but where the most successful projects are often interrupted by death from pestilential influences, appear to plunge a large proportion of the population into what may be termed the excesses of devotional feeling, whilst the understanding of the young is lavishly over-exercised. It is the object of Dr. Brigham's work upon the Influence of Religion to point out these excesses, but not to discourage devotion; to shew the evil consequences of fanaticism, but not to disparage a pure and simple and earnest worshipping of the Deity.

Yet we believe this attempt to detach an enthusiastic people from extravagant and hurtful customs, has brought upon the author much obloquy; as if to expose the follies which degrade religion were to discountenance piety itself.

In the history of mankind there is nothing that creates more surprise than the contemplation of the cruelties which have been practised by various nations, in every part of the globe, with a view of propitiating the Almighty. These cruelties have been, and are, partly physical and partly moral, and, although their most revolting features have disappeared, the spirit which suggested them, full of violence and unenlightened, has not yet been subdued by the milder doctrines which Butler has termed the *republication* of the morality of nature. When we read the religious history of an ancient people, or the religious wars of later ages, it is difficult to believe that we are reading the history of beings capable of humanity and justice. That men who felt themselves, in simple or savage state, dependent on some unseen power which they could not resist, should have endeavoured to propitiate that power by the same acts with which they soothed the angry passions of each other, is not to be wondered at; and if they fell into the error of believing acts of horror acceptable to that power, we derive from the fact a most useful lesson. We learn that the *uninstructed* man cannot even read the true character of the Deity; that the works of nature address their eloquent language to him in vain; that the destiny of man is obscurely beheld by him; and that it is only as he advances in knowledge that he becomes *capable* of receiving and comprehending divine truths, abandons ceremonies stained with blood, and tries to imitate the goodness which he then alone discerns in action all around him.

Dr. Brigham's book presents us with a brief and distinct view of much of this extraordinary part of man's history. He commences by noticing human sacrifices, as detailed by the historians of Egypt, Scythia, Persia, Greece, Rome, Gaul, Germany, and the northern nations, and by the explorers of the Eastern, Southern, and Western portions of the globe. He passes from these dreadful details to a notice of the mutilations of the body which have been, at various times and in various regions, practised with the same view of obtaining merit in the eyes of the Creator; such as circumcision, emasculation, flagellation, wounds voluntarily inflicted, and deformity (anchylosis of joints) voluntarily incurred. To the consideration of these succeeds that of the milder austerities of penance and fasting; and this review introduces reflections marked by much moderation and good sense, on the customs still prevalent which, altogether unessential to the purity, sincerity, and even earnestness of religion, bring inconvenience and suffering upon conscientious and devout persons. We have not often perused any thing more interesting than these details; the worst enormities of which, in remote periods, are attested by the customs actually existing in the South Sea Islands, and even in Hindostan; whilst the peculiar character of the superstitions of the most civilized states of America is forcibly represented to us by a witness who cannot be accused of indulging national prejudices at the expense of truth.

Among the various modes of self-torture which men and sects have at different periods assumed to be pleasing to a benevolent Creator, that of *flagellation* seems to have been not the least remarkable. The severe whippings inflicted upon the boys of Sparta, before the altar of Diana, had for their intention the propitiation of the goddess. It was reckoned honorable to make no complaint, and the victims of this superstition sometimes expired under the lash. But a more celebrated sect of *flagellants* appeared in Italy, about the middle of the 13th century: they ran about with whips in their hands, lashing themselves severely. In the next century they increased so much in numbers and turbulence that at length a holy war was declared against them; 8000 were massacred, and the remainder led away captive. Nor has such foolish cruelty been without modern imitators, and even in the State of New York; where, Dr. Brigham tells us, parents whipped their children as a religious duty, and to make them "submit themselves to God." A pious lady in Oneida county is quoted as having whipped all or most of her children by way of *bringing them in*, by which is meant *converting* them. One of them required whipping three times. A worthy of the Oneida presbytery, looked upon as a great authority in religious matters, and "whose praise is in all the churches," being asked to give his opinion of the propriety of "whipping children, to induce them to promise to give themselves to God," replied that he thought there was "much to be said in favour of it," and that children were rendered "kind and affectionate" by it. One cannot but wish that the process had been tried upon this unkind bigot himself. From a like spirit arose many of the gloomy austerities of monachism. Man was perpetually represented as a criminal, to whom cheerfulness was forbidden, and by whom gaiety and laughter were to be utterly forsworn. Sickness was courted, as natural to a Christian; and the doctrines of physicians, which taught men how to preserve health and life, were despised or condemned, as hostile to the very spirit of Christianity.

We pass over the author's remarks on Fasting, on the mode of celebrating the Lord's supper, and on Baptism; as involving many controversial questions, unsuitable to our pages. With rare exceptions none of these ordinances are so practised in this country or in other countries as to affect the health, nor are they, we presume, precisely the ceremonies most likely to be practised with detriment in the United States. But the subject of Dr. Brigham's Fifth Chapter will, we hope, attract attention among his countrymen; comprehending, as it does, a consideration of the places of worship, and the night meetings and camp meetings held among the Americans.

"There is not perhaps anything more beautiful in the scenery of New England, than the churches and spires that are seen in almost every town. They are generally built of wood, painted white, and impress the traveller with favorable ideas of the order and piety of the inhabitants around. I wish I could say that these churches are as comfortable for worshippers, as they are beautiful to the observer; but in truth they are not. In general they are poorly built, and badly keep out the cold of winter and the heat of summer. The seats, usually unsupplied with cushions, are very uncomfortable places to remain in, even for two or three hours. Many of these churches are placed upon the tops of hills, where they are exposed to the violence of wind and cold, unprotected by woods or rising grounds. They have neither inside nor outside shutters to the windows, and as they are greatly lighted, the heat of a summer's sun is exceedingly oppressive.

"But this can be better endured than the cold of winter. Within a very few years, however, this evil has been greatly lessened, and many churches have had stoves placed in them, and are partially warmed; but, even now, I presume one quarter of the churches in New England, in the country towns, are destitute of any means of being warmed, and those thus unsupplied are the churches situated in the most bleak and cold places in the country. The suffering from this cause is great, and many lives, I have no doubt, have been sacrificed in consequence." (P. 139.)

The churches of our own country are not exempt from some of these inconveniences. They are ill ventilated, very cold in winter, and very oppressive in summer; and are often, in our opinion, the unsuspected causes of attacks of illness in delicate persons.

Most of the Calvinistic, methodist, and baptist churches in New England, and in the northern and middle states, have, it appears, two, three, or more meetings in the evening every week; besides which there are evening meetings for prayer, and in aid of numerous charitable and religious societies; and evening conferences once a week or oftener in most of the churches; so that Dr. Brigham says more than one-half of the evenings of the year are thus employed; and some of the zealous attendants, especially females, occupy in this way every evening in the week,—a practice which he justly condemns, as "encouraging a kind of theatre-going spirit, i. e. a love of excitement, incompatible with a love of domestic life and patient study and research at home." In some of them he has noticed a disturbance of health, and a tendency to nervous diseases, thus created. The excitement itself he considers to be useless as well as dangerous. "I have known people," he says, "all anxiety to hear a man who had visited China or some other country, give a lecture describing the places he had visited; yet these persons had never taken pains to inform themselves respecting those countries, by reading any of the full and authentic accounts of them, to be found in numerous books." We might find excuses for such a pardonable curiosity; but the case becomes serious when, as we learn from Dr. Brigham, throughout the whole community at least twenty-five, if not fifty, out of one hundred females, attend religious meetings at least 100 or 150 nights in a year.

The camp-meetings of America have been described by travellers, and sometimes probably with exaggerations, in books that are in everybody's hands. Dr. Brigham gives an account of them, and even of the fanatical language and practices by which they are characterized, with his usual moderation, setting forth the facts, often in the very words of the performers themselves, but indulging in few observations, and those free from unnecessary severity. In all these instances it is but proper to give the devotees the credit of sincerity, and the sincerity of piety deserves respect. But the consequences of such feelings, when unrestrained by a calm judgment, are generally of a nature to be deplored; and the camp-meetings unfortunately seem to furnish exhibitions equally condemned by the judgment and by a due sense of propriety. They are meetings held out of doors, and usually in the woods, for several days and nights in succession. Thousands of individuals attend them, and the professed object is a devotion of themselves during the whole time to prayer and other religious exercises. The accounts of these meetings, written by the ministers, abound in narrations of wonderful awakenings and conversions, some of which it is difficult to read without a smile.

As regards the subject of health, it is sufficiently evident that the exposure to the weather, either in the open air or in tents, during such camp-meetings, must be highly injurious to many who attend: but the ill-judged exhortations of the preachers, the frantic demeanour of the converted, the tears and groans and wild excitement of the multitude, produce in many cases the most pernicious impression on the minds of the young, the delicate, and the susceptible; sometimes ending in absolute insanity. The effect upon the preachers themselves appears to be, in almost every instance, an early breaking down of the constitution; and it has been remarked by close observers, that the general results upon the congregation are similar to those which would be produced by strong drink, a temporary and delusive appearance of strength, followed by sensorial debility and disease.

If any one feature of these fanatical proceedings were selected as more melancholy than the rest, it would be the effect of similar impressions made on children. Dr. Brigham quotes some distressing examples of the state of mind and body produced by constant and intemperate efforts to produce miracles of early piety. Such efforts seem as destructive as similar attempts to produce paragons of early mental accomplishment. In both cases the brain, or some portion of the nervous system, is overwrought, and disease, delirium, and early death are the common consequences. It is impossible to read the case of Fanny Harrison, related with self-righteous complacency by the by-standers, (p. 178,) without the most painful feelings. An interesting child of eleven years of age is stated *only then* to have become "anxious about her soul." Still, "she had not submitted her heart to God." Attendance on the protracted prayer meetings, and the reproval of all cheerfulness at home, seem to have caused the poor child to be affected with fever, of which she died. We forbear to dwell upon these topics, on which it would be really difficult to express one's feelings with calmness. But such facts prepare us fully to believe the assertion of several observers quoted by Dr. Brigham, that nervous disorders of all kinds, including complete derangement of mind, are becoming more and more prevalent in America. We trust, however, that his work, whatever prejudices it may have offended, will excite such a degree of attention as may check the unreasonable ostentation of devotion which now appears so much to prevail in most of the states. The excitement of mind incidental to a people so jealous of their freedom, and at the same time so indefatigable in the pursuit of wealth, might be mitigated by a calm devotional spirit; whereas the extravagant demonstrations of religious enthusiasm, whilst they further affect and agitate the mind, are, we fear, too often put in the place of an even course of correct conduct, and a due government of worldly desires.

To all who are deeply engaged in the pursuit of the objects of ambition, whether power or possessions, the Eighth Chapter of Dr. Brigham's work may be recommended for careful perusal; and those who in simplicity and earnestness of heart have devoted themselves to religious exercises of which they already begin to feel the debilitating effects, will find much in it deserving of their most serious attention. They will see how directly the brain and nervous system are affected by all vehement emotions, and how variously and seriously the body becomes injured by this nervous disturbance; and surely it will then only require the exercise of reason to convince them that the Creator, whose bounty endowed the body and mind with so many faculties, does not demand the premature destruction of those faculties as a religious duty. To suppose that excitements, leading to exhaustion and religious melancholy, often ending in suicide, and sometimes in murder, can be pleasing to the great and benevolent Creator, is to admit an opinion unworthy of a rational and instructed people, and utterly inconsistent with the character of the Deity, either as revealed in the holy writings or in the great book of nature.

From these subjects, however, the fuller discussion of which does not form a part of our duty in this work, we turn to Dr. Brigham's publication on the Influence of Mental Cultivation and Mental Excitement upon Health, written a few years earlier, and the appearance of which seems to have been occasioned by his observing the injudicious attempts of parents in general, throughout the United States, to cultivate the minds of their children, at an early age, to an extent quite unsuited to their years and strength, and productive of the worst effects. We are glad to see, by the preface to the second edition, that a conviction of these errors begins to gain ground; although Dr. Brigham is still of opinion that there is much injury caused by too much mental labour being required of children, and by the repression of their natural gaiety and love of amusements. In the preface to the first edition, Dr. Brigham remarked that the females of the United States, especially those in cities, were in general more delicate and feeble than those of Europe; and, at the same time, that there was no country where females received such early and great intellectual culture, and had so little attention paid to their physical education. It appears, too, that in consequence of the openness of the road to the highest honours for those who evince a capability of serving the republic, efforts, much more strenuous than judicious, are systematically made to exercise and store the minds of children. Infants' manuals of all kinds are sold at the book-stores, on various subjects of science, and new and short methods of universal instruction are sure to find many patrons.

Addressing a population prone to such fatal mistakes in the management of children and young persons, Dr. Brigham devotes several chapters to explanations not required by medical readers, but which are extremely well placed in a work intended to inform the public. He points out the reasons which have induced physiologists to consider the brain as the material organ by which the mental faculties are manifested, describes the condition of the brain in early life, and the known effects of its excitement. He urges that mental precocity is usually a symptom of disease, and not a promise of future distinction, and adduces examples to prove that the best and most powerful minds have not been formed by early culture carried to excess; and in support of these opinions he quotes many authorities.

The section of this little work which is calculated to make the strongest impression on his readers, is that in which he shews the influence of mental cultivation and mental excitement in producing insanity, nervous affections, and diseases of the heart. He states that the number of individuals deranged in mind in the United States is as 1 to 262 of the inhabitants, whereas in England it is but 1 in 820, and in Scotland 1 in 574. The moral causes of this great excess in America deserve investigation; and there can be little doubt that the greater number of them would be resolved into some excitement of the brain. It is an undoubted fact that insanity prevails most in countries where the mind is allowed the freest exercise, and where the people enjoy the greatest extent of political liberty. It is common in America, in England, and in France; rare in Russia, in Spain, in Turkey, and in China. Among the educated it is most prevalent in those whose imagination is most indulged, and whose reasoning powers are least exercised. In all times of national commotion it is more than usually common. It is rare in children and young persons, but is sometimes observed in them as the result of violent emotions, or of excessive application to study. Dr. Brigham ascribes its extraordinary frequency in America to four principal causes; 1, too constant and too powerful excitement of the mind, which the strife for wealth, office, political distinction, and party success, produces in that free country; 2, the predominance given to the nervous system, by two early cultivating the mind and exciting the feelings of children; 3, neglect of physical education, or the equal and proper development of all the organs of the body; 4, the general and powerful excitement of the female mind.

We are certainly not among those who consider the population of Great Britain as being yet over-educated; but it cannot be denied that even education may be pushed, or perverted, to the extent of pernicious excitement. There are also evils incidental to education, which are erroneously supposed to belong of necessity to education, but which are only the product of prejudices not yet worn out. Among these may be mentioned the long-continued confinement of children in charity and Sunday schools; in consequence of which, many poor children, without acquiring the blessings of real knowledge, incur the evils of a wearied or over-excited brain. Some of these evils are strikingly set forth in Dr. Brigham's statements respecting the city of Hartford, in the United States, of which the population is not more than seven thousand. Nearly all the children commence attendance at school as early as the age of three or four, and attend six hours a day. Many of them attend Sabbath schools, and go twice to church on Sundays, being thus occupied altogether about six hours. There are nine large churches in that little city, well filled twice or thrice every Sunday; and meetings, twenty or thirty times a week, in some or other of these, on other days. There are two literary associations, where meetings are held once a week, open to all; at one of which lectures are given, whilst debates are held on political or historical subjects at the other. *Seven* large weekly newspapers are published, and *five* large religious papers. Several other periodical works are published at Hartford; and the papers and reviews of the other cities are eagerly read, and most of the English reviews and magazines are taken. These circumstances assuredly bespeak an astonishing degree of mental activity in so small a population; and it is a most serious question for the legislator to consider, whether it is in a direction to promote health and happiness, or whether, as Dr. Brigham apprehends, these multiplied means of stimulating the mind are slowly producing effects to be transmitted to another generation; not an increase of intellectual power, but increased susceptibility of the brain and nervous system; individual disease, and national degeneracy.

These observations imply no distrust of the benefit, not even a shadow of doubt concerning the positive duty, of educating every rational being; but the idea of education must not be limited to the mere exercising and storing of the mind. A complete education includes attention to the emotions and the moral feelings, and its objects are individual happiness and the general good; objects which unfortunately continue to be too much overlooked. Education is desired as the means of making a fortune, and of attaining a certain station; not as a means of securing simple independence, contentment, a tranquil mind, a habit of benevolence, well-governed affections, and the power of being useful to others. If, then, health is sacrificed, if ambitious views are disappointed, and morality not increased, it is unjust to cry out against education. A partial and unwise system of education produces but partial benefit; a more complete and wiser system would extend it; and such a system would discountenance and exterminate the evils of the false and worldly system to which the name of education is most improperly given. Then it would be found that the proper education of the body was favorable to the healthy condition of the mind; and a rational and cheerful exercise of the mind promotive of health of body; and that both were favorable to the happier emotions of which the frame is susceptible, and which are often annihilated by the destructive struggles made for worldly and selfish advantages.

Dr. Brigham's concluding chapter is chiefly devoted to the illustration of an opinion which deserves to be attentively weighed not in America only, but in Europe; namely that dyspepsia, far from being invariably a disease of the stomach, is often the result of a disturbed and irritated brain. We are ourselves convinced from observation that what are called sick headachs, occurring in irritable individuals, are often the mere product of disturbance of the nervous system. Nor do we in the least degree doubt that in this busy age, a very great proportion of the cases of indigestion, debility, and nervous pains of which individuals complain, are but the effects of too much labour of the brain; and we entirely go along with the author in believing that the most careful diet will fail to relieve many forms of indigestion unless the existing pressure and irritation can be removed from the mind. This view is entirely borne out, too, by the fact, that comfortable living and freedom from care are of all circumstances the most favorable to longevity. Of these truths we trust the readers of Dr. Brigham's very useful works will become convinced to their advantage. We are truly glad to see the medical men of the United States stepping boldly forward, in the face of many prejudices, to instruct the public concerning their real good. It has ever been the just praise of the profession that its members have done so; and those who cultivate medicine in the United States seem actuated by the same desire to do their duty, and at the same time fully capable of performing that duty efficiently.

---

